# Efficacy of Beetroot Juice in Patients With Peripheral Artery Disease: A Systematic Review

**DOI:** 10.7759/cureus.88590

**Published:** 2025-07-23

**Authors:** Carlos A Umaña Mejia, Yolanda García Aguilar, Yolik N Carracedo González, Victor F Nuñez Lara, María F Miranda Corona, Wendy M López Moreno, Carlos Rodríguez Abrego, José de Jesús Tabares Montiel, Daniel A Medina Lam, Katia Guevara Cortés, Alonso E Vallejo Risueño, María J Herrera Yáñez, Jaqueline L Castillo, Jose R Flores Valdés

**Affiliations:** 1 General Medicine, Universidad Autonoma De Guadalajara, Guadalajara, MEX; 2 General Medicine, Universidad Autónoma de Zacatecas, Zacatecas, MEX; 3 General Medicine, Universidad de Guanajuato, Guanajuato, MEX; 4 General Medicine, Universidad Marista de Mérida, Mérida, MEX; 5 General Medicine, Universidad Anáhuac Querétaro, Querétaro, MEX; 6 General Medicine, Universidad de Ciencias Médicas,, San José, CRI; 7 General Medicine, Instituto Tecnologico de Monterrey, Zapopan, MEX; 8 General Medicine, Universidad Veracruzana, Veracruz, MEX; 9 General Medicine, Universidad de Guayaquil, Guayas, ECU; 10 General Medicine, Universidad Autónoma de Sinaloa, Sinaloa, MEX; 11 General Practice, Instituto de Cardiología Preventiva, Querétaro, MEX; 12 General Medicine, Universidad Autonoma de Guadalajara, Guadalajara, MEX; 13 General Medicine, Oncology Consultants, P.A., Houston, USA

**Keywords:** beetroot juice, blood pressure, claudication, dietary nitrate, exercise capacity, nitric oxide, peripheral artery disease, randomized controlled trials, walking distance

## Abstract

Peripheral artery disease (PAD) is a prevalent manifestation of atherosclerosis, characterized by reduced blood flow to the lower extremities and associated with claudication, pain, and limited exercise capacity. Nitric oxide (NO) plays an essential role in vascular homeostasis, and beetroot juice (BRJ) is a natural source of dietary nitrate that has emerged as a potential therapeutic option for improving vascular function and exercise tolerance in PAD. This systematic review followed the Preferred Reporting Items for Systematic Reviews and Meta-Analyses (PRISMA) 2020 guidelines, including a flow diagram, a checklist, and reporting items, and was registered in PROSPERO (number CRD420251059989). We searched PubMed and ScienceDirect from inception to February 2025 for randomized controlled trials (RCTs), cohort, and case-control studies evaluating the effects of BRJ in adults (>18 years) with stable PAD. Inclusion required a confirmed diagnosis of PAD (ankle-brachial index <0.9) and history of intermittent claudication. Outcomes included claudication onset time (COT), peak walking time (PWT), and blood pressure changes. Risk of bias was assessed using the Cochrane Risk of Bias tool. From an initial yield of 315 articles, five RCTs met the inclusion criteria, encompassing a total of only 64 patients (age range: 52-80 years; 57.8% male). Four of five trials utilized crossover designs and one was a parallel RCT, all comparing BRJ to placebo over short follow-up periods ranging from one to 84 days, which contributed to significant heterogeneity in outcomes. Two studies demonstrated significant differences in COT and PWT, while others reported either no significant changes or improvements in both groups. Reductions in systolic and diastolic blood pressure were observed in three studies, and two reported increased serum nitrate/nitrite levels following BRJ intake. No adverse events were reported. In conclusion, while beetroot juice intake may offer potential benefits such as enhanced exercise capacity and reduced blood pressure in PAD patients, these findings are based on preliminary evidence from a small number of studies with limited sample sizes and varying methodologies. The proposed mechanism of action via nitric oxide bioavailability remains a hypothesis, not directly confirmed by the included trials. Given the moderate-to-high risk of bias, small study populations, and heterogeneity in dosing, duration, and outcome definitions, these results should be interpreted cautiously. Larger, long-term randomized trials with standardized protocols are warranted to clarify its clinical utility and support its incorporation into therapeutic strategies for PAD.

## Introduction and background

Peripheral artery disease (PAD) is a form of cardiovascular disease, recognized as a manifestation of atherosclerosis in the peripheral vascular system, mainly in the lower extremities. It is attributable to the accumulation of plaques in the arteries, causing narrowing or progressive blockage of them [1-2]. This leads to a decline in arterial perfusion of the lower extremities, which can manifest as calf pain during periods of increased demand, such as with exertion or walking, which is commonly relieved by rest, also known as claudication [3].

PAD is a condition that can manifest differently in many patients; some can have an asymptomatic disease, while others present with a mild, moderate, or severe disease, such as limb-threatening ischemia, nonhealing ulcers, and gangrene [3]. A significant proportion of PAD cases remain undiagnosed due to variable clinical presentation. Nonetheless, it is estimated that around the world, over 200 million adults are affected by this condition [3], 8.5 to 12 million of whom live in the United States [1].

As a manifestation of atherosclerosis, it is associated with risk factors such as cigarette smoking, diabetes mellitus, high total cholesterol, and hypertension. Owing to that, PAD correlates with significant cardiovascular morbidity and mortality, and that is one of the reasons why its recognition is especially important [2]. The diagnosis can be made by taking a detailed medical history, performing a meticulous examination, and taking into account the results of different tests or procedures, such as the ankle brachial index (ABI) test, which is one of the first and most used [1,3].

The management of this condition involves improving symptoms and quality of life and, due to its pathophysiology, reducing the risk of cardiovascular and limb events. Current guideline recommendations include different approaches such as supervised exercise training and endovascular revascularization depending on patients' particular characteristics [2]. Other suggested treatments include cilostazol and naftidrofuryl, which are said to be used in patients with intermittent claudication but with substantially limited quality of life and who cannot participate in exercise programs. Regardless, these medications have shown limited efficacy and frequently present with side effects [4]. Therefore, even with all these treatment strategies, many patients still experience major debilitating symptoms [5]. Having said that, it becomes clear the need for potentially beneficial therapies with higher efficacy and lower side effect profiles. 

Nitric oxide (NO) is a molecule that participates in the regulation of vascular tone by its action in smooth muscle [5]. While in healthy individuals shear stress in the vasculature stimulates the production of NO by the endothelium, in patients with PAD, this mechanism is compromised. Endothelial dysfunction and oxidative stress in patients with PAD prevent NO augmentation in this scenario, leading to low flow-mediated dilation of the affected vasculature. Due to that, treatments focused on increasing NO levels in these patients might improve pain with exertion and other symptoms by promoting revascularization in the lower limbs [4,6]. 

Different interventions can be used to increase NO levels in patients, one of them being the use of dietary nitrates and nitrites, which can be reduced to NO [4]. Beetroot juice (BRJ) is known to contain great quantities of nitrate, which can then be converted to nitrite and eventually to NO. Patients with PAD in whom hypoperfusion leads to a particularly ischemic environment have increased conversion of nitrites to NO [7]. Randomized clinical trials (RCTs) have been conducted to prove that dietary supplementation of BRJ positively impacts PAD patients' symptoms by measuring different criteria, such as claudication onset time and walking distance. The main objective of this systematic review is to assess whether BRJ improves exercise capacity in PAD patients by evaluating outcomes such as claudication onset time and maximal walking distance. 

## Review

Methods

This study adhered to the Preferred Reporting Items for Systematic Reviews and Meta-Analyses (PRISMA) 2020 guidelines and followed principles of evidence-based medicine to ensure a comprehensive and systematic approach to the review [8,9]. The protocol was registered in the PROSPERO database (registration number: CRD420251059989). 

*Search Methods  * 

To ensure the inclusion of only robust and relevant studies, strict eligibility criteria were applied. Studies lacking methodological rigor or not meeting predefined standards were excluded. Furthermore, articles without accessible full texts, including those unobtainable through interlibrary loan services, were also omitted. 

A comprehensive literature search was carried out using databases such as PubMed and ScienceDirect. The search protocol incorporated a combination of Medical Subject Headings (MeSH) and pertinent free-text keywords tailored to the research objectives. A targeted search strategy was employed, incorporating relevant terms such as "beetroot juice", "NO₃⁻", and "peripheral artery disease". Study selection followed a structured process outlined by a PRISMA flow diagram. This thorough methodology contributed to the assembly of a consistent dataset, supporting the reliability and precision of the final analysis. 

Selection Criteria  

Types of participants: For this systematic review, we established selection criteria that included studies of patients aged ≥18 years, men and women, regardless of race, with a diagnosis of PAD and with a history of stable intermittent claudication. We excluded studies that involved patients with ischemic rest pain or tissue loss due to PAD, limited walking ability due to conditions other than PAD, and patients who regularly consumed a form of NO3− (foods or supplements high in dietary nitrate).  

Types of intervention: This systematic review focuses on therapy with BRJ versus placebo in patients with PAD. 

Types of studies: A systematic search was conducted for relevant research articles published in English from the inception of each database up to February 2025. The selection focused on studies that met specific inclusion parameters, namely clinical trials, cohort studies, and case-control studies. To uphold the rigor of the analysis, various publication types were excluded, including case reports, expert commentaries, case series, letters, cross-sectional studies, book chapters, study protocols, conference abstracts, and previously published systematic reviews or meta-analyses. 

Type of outcomes: The primary outcomes of this review are to assess the effect of dietary nitrate supplementation in the form of BRJ versus placebo on pain-free/claudication-free walking distance reported in meters or seconds, maximum walking distance/time reported in meters or time values. Secondary outcomes include changes in blood pressure, exercise capacity, and serum nitrate/nitrite concentrations. 

Selection of Studies, Data Extraction, and Screening  

The initial screening of article titles and abstracts was independently conducted by two reviewers (AEVR and MJHY) using the Rayyan platform [10]. To ensure consistency with the eligibility framework, a third reviewer (MFMC) reassessed the screened records for alignment with the predefined inclusion and exclusion parameters. After this preliminary stage, full-text documents were carefully examined. Two additional reviewers (YGA and CRA) independently evaluated the selected studies using the same eligibility benchmarks. Any discrepancies encountered during this phase were addressed through discussion and, when necessary, resolved with input from a third reviewer (CAUM). Relevant data, such as sample size, age, BRJ dose, and follow-up time, were then systematically extracted from the studies that met the final inclusion criteria. 

*Data Evaluation: Assessment of Risk of Bias  * 

The assessment process was guided by the methodological standards provided in the Cochrane Handbook [11]. For RCTs with crossover, the Cochrane Risk of Bias tool for randomized crossover trials was used [12], and for RCTs, the Cochrane Risk of Bias tool [13]. Two reviewers (KGC and YGA) independently evaluated the potential risk of bias for each included study, adhering to the criteria and recommendations specific to the tools applied. Any differences in judgment were discussed and reconciled with the involvement of a third reviewer (CRA). Modifications to the certainty of the evidence - whether downgrading or upgrading - were clearly documented and reported in the summary of findings table 

Results  

A structured literature review was undertaken to assess the effects of BRJ supplementation in individuals diagnosed with PAD, specifically those experiencing stable claudication. The search spanned from the inception of the selected databases up to February 2025 and was conducted using platforms such as PubMed and ScienceDirect. A targeted search strategy was employed, incorporating relevant terms such as "beetroot juice", "NO₃⁻", and "peripheral artery disease". 

The initial search retrieved 315 records. After removing duplicates, 281 unique articles remained. Titles and abstracts were screened systematically, resulting in the exclusion of 271 articles. Of the remaining 10 records, full-text access was secured for nine, while one could not be obtained despite attempts. Further full-text evaluation led to the inclusion of four studies. An additional eligible article was identified through manual citation tracking, bringing the final total to five studies that fulfilled all predetermined eligibility criteria. 

**Figure 1:**
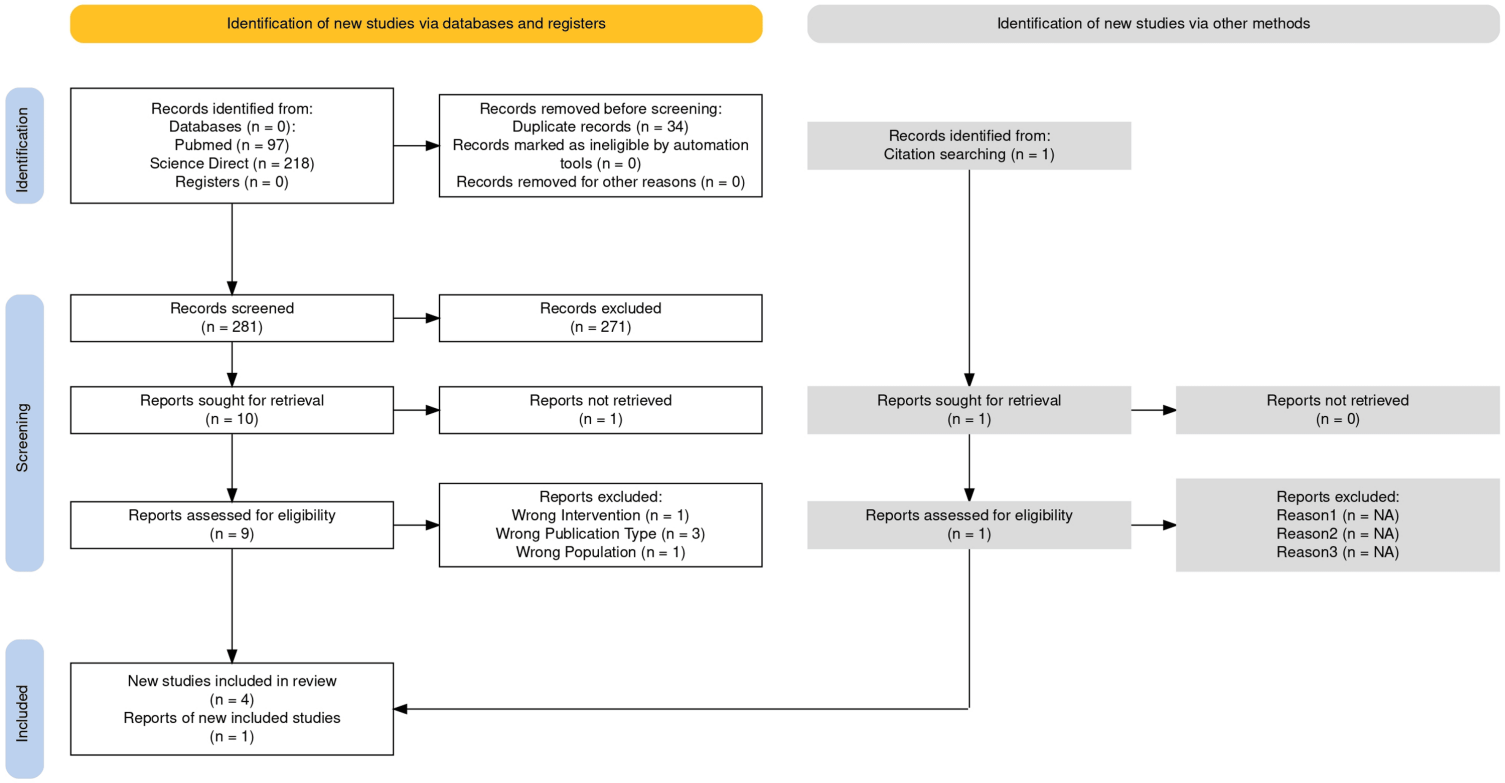
Flow diagram of bibliographic search This diagram details the step-by-step process of article screening [8,14].

The selection workflow is visually represented in Figure 1, structured in accordance with the PRISMA flow diagram [8,14]. This diagrammatic representation enhances the transparency and reproducibility of the screening and selection process. 

**Figure 2:**
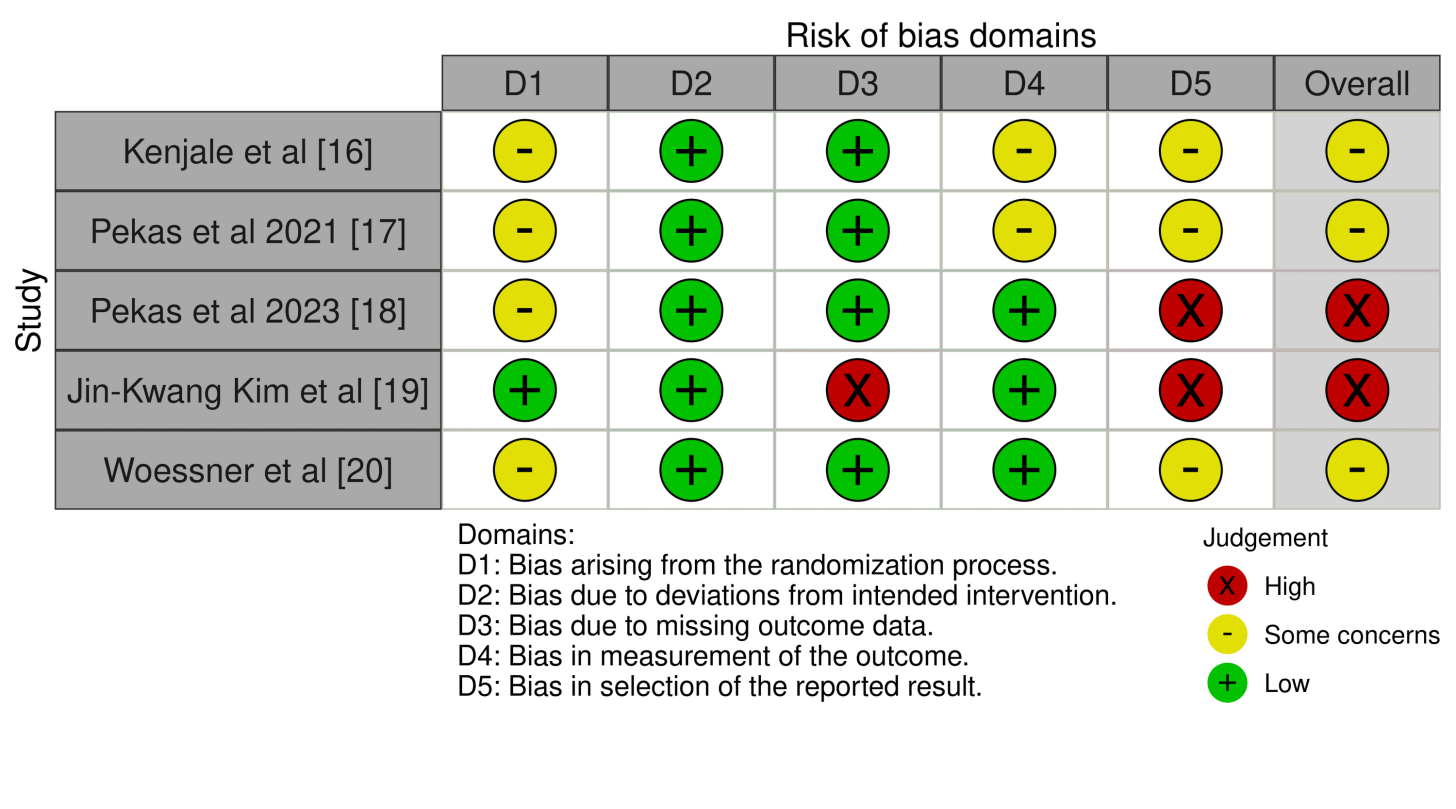
Risk of bias Each article was assessed for risk of bias [12,13,15].

**Figure 3:**
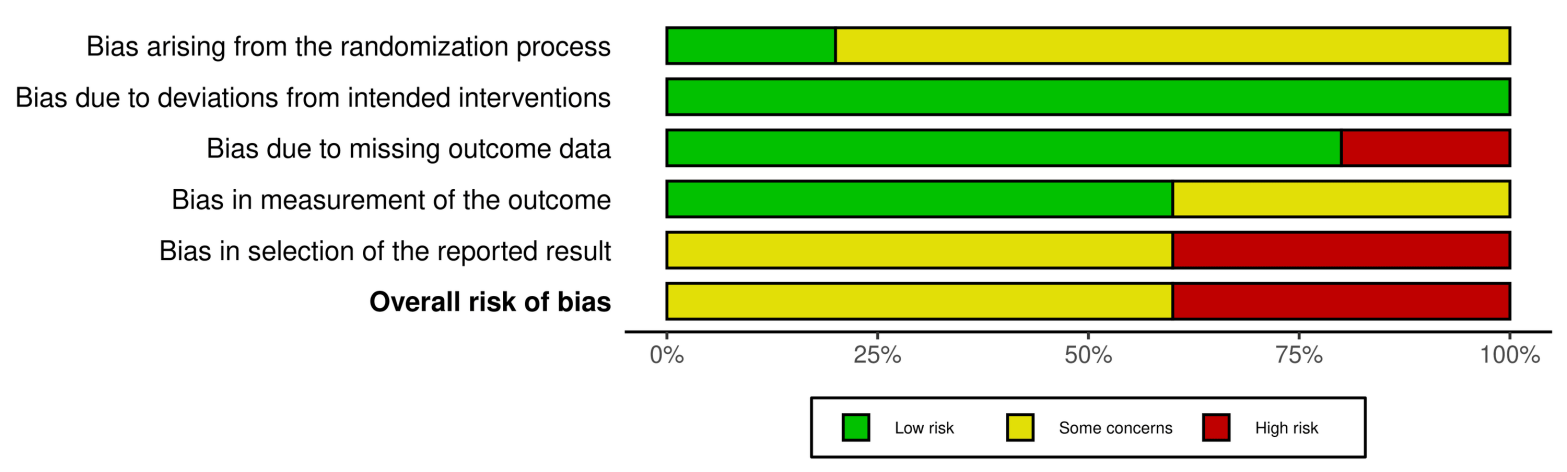
Summary plot for risk of bias This diagram represents the risk of bias in a summarized way [12,13,15].

Our risk-of-bias assessment, presented in Figures 2-3, indicates that the most common possible source of bias in the included studies was in the selection of the reported results, with three out of five studies with some concerns and two out of five with high risk in this domain. The next common source of bias was about arising from the randomization process, given that four trials had some concerns about bias, and one had a low risk. All the studies included had a low risk of deviations from the intended intervention. About bias due to missing outcome data, just one study had a high risk, and the other four low risk. The risk of bias in the measurement of the outcome was moderate in two trials and low in three. 

Five studies fulfilled our inclusion criteria, giving a total of 64 patients, with an age range from 52 to 80 years, and 37 (57.8%) were male. All trials included were performed in the United States. Four of the five studies included were crossover RCTs, and one was a double-blinded RCT. Every participant had the diagnosis of PAD (ABI <0.9) and a history of stable claudication. In the intervention, two trials used 70 ml of BR juice, two an average of 90 ml, and one used 500 ml; all of them were compared against the use of a placebo. The follow-up time was 14 days in three trials, one for 84 days, and the other was performed in a single day. No adverse events were reported in the studies.  The difference in dosing and follow-up time across trials adds to the heterogeneity when evaluating outcomes. Importantly, crossover trials may have limitations such as restricted application for long-term outcome measurement and possible carryover effects. 

Results of individual studies 

Regarding the onset of claudication pain (COT), Kenjale (2011) reported an increase of 32-s (18%) (p ≤ 0.01) and 65-s (17%)(p ≤ 0.05) increase in peak walking time (PWT) with the consumption of BR compared with PL [16]. Parallelly, Pekas (2021) presented an increase in the maximal walking distance by (D92.7 ± 36.8 m, P = 0.029) [17], and Pekas (2023) reported that six-minute walking distance was greater after BRJ intake compared with placebo (PL: Δ −7.3 ± 10.7 m, BRJ: Δ 37.5 ± 9.1 m, p = 0.007), but there was no change in pain-free walking distance (PL: Δ −1.6 ± 12.7 m, BRJ: Δ 53.1 ± 17.7 m, p = 0.133) [18]. On the contrary. Jin-Kwang Kim (2024) did not find a difference between the placebo and BR nitrate in the PWT (BRplacebo vs. BRnitrate; 691.3 ± 547.7 vs. 730.5 ± 604.5 s; p = 0.473; Cohen’s d = 0.068) and COT (BRplacebo vs. BRnitrate; 195.2 ± 136.1 vs. 200.2 ± 149.7 s; p = 0.41; Cohen’s d = 0.035) [19], and similarly, Woessner (2018) reported a related pattern in the PL and BR in the six-minute walk of (24.6 ± 12.1 and 53.4 ± 19.6 m) (p ≤ 0.05) 0.43 (95% CI, −0.44 to +1.21) and PWT (238.7 ± 207.0 and 269.9 ± 195.3 s) (p ≤ 0.01), since both of them increased significantly for both groups [20]. As secondary outcomes evaluated in our systematic review, Kenjale et al. and Jin-Kwang Kim et al. reported a significantly decreased diastolic blood pressure (DBP) post-beverage ingestion of BR in contrast to PL (p ≤ 0.05) [16,19]. In addition, Pekas (2021) reported that the central systolic BP was reduced (D 8.2 ± 3.1mmHg, p = 0.014) after NO3 intake, contrary to PL [17]. Regarding the serum nitrate/nitrite concentrations, two trials found a significantly increase after NO3 intake compared with placebo (p ≤ 0.05) [16,17], and Woessner (2018) did not find differences in plasma nitrate and nitrite concentrations neither at the beginning or at the end of the 12-week study [20]. It should be noted, however, that the included studies reported COT, PWT, and walking distances in different units (seconds vs. meters vs. Δ‐values), and this adds heterogeneity across studies. Comparisons between values are form baseline results in patients. Table 1 summarizes all information in an organized manner. 

**Table 1:** General outcomes RCT: randomized controlled trial, F/M: female/male, NO3: nitrate, BP: blood pressure, SBP: systolic blood pressure, PAD: peripheral artery disease, COT: claudication onset time, PWT: peak walking time, Δ: mean change

Author, year	Kenjale et al., 2011 [16]	Woessner et al., 2018 [20]	Pekas et al., 2021 [17]	Pekas et al., 2023 [18]	Jin-Kwang Kim et al., [19]
Study type	Randomized open-label crossover study	RCT double-blind	RCT double-blind crossover	RCT double-blind crossover	RCT double-blind crossover
# of total patients in study	8	24	11	10	11
# of patients intervention group	4	11	11	10	11
# of patients placebo group	4	13	11	10	11
Beetroot juice dose	500 ml	4.2 mmol NO3-	0.11 mmol NO3- /kg (91.8 ± 20.0mL)	0.11 mmol NO3- /kg (90.0 ± 20.2mL)	70 ml (0.3 g inorganic nitratre) twice daily
Mean age of the intervention group	67 ± 13	67.5 ± 8.6	70.0 ± 7.0	67.9 ± 8.7	64.5± 8.0
Mean age of the placebo group	67 ± 13	71.5±7.3	70.0 ± 7.0	67.9 ± 8.7	64.5± 8.0
# of F/M in the intervention group	2/2	3/9	6/5	6/4	9/2
# of F/M in the placebo group	2/2	7/6	6/5	6/4	9/2
Race or ethnic group # (%) intervention group	Caucasian: 3 African-American: 5	White: 8 (72.7) Black: 2 (18.2) Asian: 1 (9.1) Hispanic: 0 (0)	N/A	N/A	N/A
Race or ethnic group # (%) placebo group	Caucasian: 3 African-American: 5	White: 7 (53.8) Black:5 (38.5) Asian: 0 (0) Hispanic: 1 (7.7)	N/A	N/A	N/A
Cardiovascular disease history and risk factors (#) intervention group	N/A	Myocardial Infarct- 2 (18.2) Stroke- 3 (27.3) Periph Intervention- 3 (27.3)	Hypertension: 8 (72.7) Dyslipidemia: 10 (90.9) Prediabetes: 3 (27.2) Current smoking: 1 (9.0) Former smoking: 4 (36.3)	N/A	N/A
Cardiovascular disease history and risk factors (#) placebo group	N/A	Myocardial Infarct- 2 (15.4) Stoke- 1 (7.6) Periph Intervention- 1 (7.6)	Hypertension: 8 (72.7) Dyslipidemia: 10 (90.9) Prediabetes: 3 (27.2) Current smoking: 1 (9.0) Former smoking: 4 (36.3)	N/A	N/A
Follow-up time	1 day	84 days	14 days	14 days	14 days
Washout time	7-14 days	N/A	14 days	14 days	7-14 days
Adverse events in the intervention group	0	0	0	0	0
Adverse events in the placebo group	0	0	0	0	0
Claudication onset time/distance intervention	215 ± 99 s	180.3 ± 46.6 s	No significant changes reported	Δ 53.1 ± 17.7 m	200.2 ± 149.7 s
Claudication onset time/distance placebo	183 ±84 s	59.2 ± 57.3 s	No significant changes reported	Δ −1.6 ± 12.7 m	195.2 ± 136.1 s
Peak walking time intervention	533 ± 233 s	269.9 ± 195.3	Not explicitly provided (Δ 56.3 s, compared to placebo)	N/A	730.5 ± 604.5 s
Peak walking time placebo	467 ± 223 s	238.7 ± 207.0	Not explicitly provided	N/A	691.3 ± 547.7 s
Maximal walking distance intervention	N/A	N/A	Not explicitly provided (Δ 92.7m, compared to placebo)	N/A (6-min walking distance: Δ 37.5 ± 9.1 m) *	N/A
Maximal walking distance placebo	N/A	N/A	Not explicitly provided	N/A (6-min walking distance: Δ -7.3 ± 10.7 m) *	N/A
Change in systolic and diastolic BP intervention	SBP: 165 ± 24 mmHg	NA	Δ -4.7 ± 1.4 / Δ -2.0 ± 1.3 mmHg	N/A	130±15 / 62 ± 9 mmHg
Change in systolic and diastolic BP placebo	SBP: 183 ± 25 mmHg	NA	Δ 1.2 ± 1.9 / Δ 0.3 ± 2.0 mmHg	N/A	140 ± 15/ 67 ± 10 mmHg
Plasma nitrate/nitrite levels intervention	Plasma NO3 reached a peak concentration at 2 h and remained elevated throughout the testing	No differences	Not explicitly provided (Δ1.32 ±0.15 μM, compared to placebo)	N/A	Plasma nitrate and nitrite concentrations increased 14 and 7-fold respectively.
Plasma nitrate/nitrite levels placebo	There were no changes in either plasma NO3	No differences	Not explicitly provided	N/A	No differences
Key points	This study shows that the consumption of a NO₃⁻-rich beverage (beetroot juice, BR) increased NO₂⁻ concentrations in plasma, which was associated with an increase in COT (Claudication Onset Time) and PWT (Peak Walking Time). This effect represents a clinically significant improvement in patient functionality, enhancing physical capacity and quality of life. Additionally, consuming a NO₃⁻-rich diet may contribute to a reduction in both diastolic (PAD) and systolic (PAS) blood pressure, further supporting its potential cardiovascular benefits.	This study shows that the exercise plus Beet intervention presented an improvement in pain tolerance. Likewise, it demonstrated a better effect on claudication onset time compared to Exercise plus Placebo. Clinically, the Exercise plus Beet intervention is beneficial for the patient instead of exercise alone.	This study shows that a body mass-normalized moderate dose of NO3- significantly improves walking capacity and skeletal muscle oxygen utilization capacity during walking in patients with PAD. It also shows it is a safe dietary strategy for improving NO bioavailability and reducing high BP in patients with PAD. All these benefits may occur in as little as 1 h after consumption of dietary NO3-.	This study design is based in the previous RCT by the same lab years earlier. The aim of this study is to show that a moderate dose of dietary nitrate can support skeletal muscle microvascular function in patients with PAD, and these skeletal muscle microvascular improvements are associated with improvements in exercise tolerance. *This study focuses on 6-min walking distance and not in maximal walking distance, showing an increase in the intervention group statistically significant compared to the placebo group.	Overall, findings suggest that inorganic nitrate supplementation in PAD patients is safe, well-tolerated, and may improve the coronary hyperemic and blood pressure responses when their calf muscles are most predisposed to ischemia.

Discussion   

PAD remains one of the leading causes of cardiovascular diseases globally, significantly increasing the risk of mortality, stroke, cardiovascular death, and myocardial infarction. Claudication remains one of the most prevalent symptoms in patients suffering from PAD, markedly reducing their quality of life and mobility. Although a variety of medications are currently available for the treatment of PAD, many come with side effects and are not completely effective in treating the symptoms. In this context, the role of BRJ, a nitrate, has emerged as a possible therapeutic agent. The main objective of this systematic review was to evaluate the efficacy of BRJ in improving symptoms such as claudication, as well as enhancing walking time and distance in this patient population. Five studies were included in the review with a total population of 64 patients. All studies utilized BRJ as the dietary supplement, with an average age range of 52-80 years. However, there was notable variation in dosage, duration of intervention (ranging from single-dose to multi-week administration), and outcome definitions across studies, contributing to clinical heterogeneity.

The findings regarding BRJ’s impact on walking capacity were mixed. Two studies, i.e., Kenjale and Pekas (2023), reported significant improvements in COT and PWT in the BRJ group compared to the placebo group. Kenjale et al., for example, observed an 18% increase in COT and a 17% increase in PWT, while Pekas et al. (2023) found a higher increase in the six-minute walking distance for the intervention group [16,18]. However, not all studies showed consistent results. Jin-Kwang Kim et al. found no significant differences between the intervention and placebo groups, and Woessner reported improvements in both the intervention and placebo groups, suggesting that the evidence regarding the effectiveness of BRJ on these parameters is still unclear [19,20]. In studies where both placebo and intervention groups improved, the possibility of a placebo effect or concurrent exercise training (even if not explicitly described) should be considered as potential confounders.

While some results, such as the 18% increase in COT reported by Kenjale et al., were statistically significant, the clinical significance of these findings remains uncertain. It is unclear whether such improvements meet established thresholds for meaningful functional gains in PAD patients, which limits interpretation of the real-world benefit.

In terms of blood pressure, the effects of BRJ also appeared promising. Both Kenjale et al. and Jin-Kwang Kim et al. found significant reductions in diastolic blood pressure in the BRJ group [16,19], while Pekas (2021) observed a decrease in central systolic blood pressure [17]. These findings are notable because they suggest that BRJ might not only improve exercise capacity but could also serve as an adjunct treatment for hypertension in PAD patients. Given that this population is at increased risk for complications like stroke and myocardial infarction, the potential for BRJ to help lower blood pressure is an important consideration. These benefits are physiologically plausible, given that dietary nitrate from BRJ is metabolized to NO, which promotes vasodilation, enhances endothelial function, and improves muscle oxygenation during exercise [4].

The small sample sizes across the included studies raise concerns about the reliability and generalizability of the results. Moreover, the short follow-up periods in these studies make it difficult to determine the long-term effects of BRJ on PAD symptoms. Four of the five studies included were crossover RCTs. In addition, three of the studies evaluated showed some concerns for bias, while two studies showed a high risk of bias. This is important as the results from these studies should be interpreted with caution. 

While the results from this systematic review suggest that BRJ may reduce claudication and improve exercise capacity in PAD patients, the evidence remains inconclusive. More research is needed, especially larger-scale, long-term RCTs, to confirm these findings and better understand the potential of BRJ as a therapeutic option for PAD. Despite the limitations of the studies reviewed, BRJ shows promise as a potentially valuable treatment for improving claudication symptoms and exercise capacity in PAD patients, but further studies are crucial to solidify its role in clinical practice.

## Conclusions

This systematic review assessed the effects of BRJ in patients with PAD, given its high inorganic nitrate content. The analysis of five RCTs (n = 64) demonstrated heterogeneous findings; some studies reported significant improvements in claudication onset time and blood pressure regulation, whereas others did not observe consistent benefits. Although preliminary evidence suggests that BRJ may be a safe adjunct therapy, likely due to its favorable influence on nitric oxide bioavailability, which may enhance exercise tolerance in individuals with PAD, these findings should be interpreted with caution. The small sample sizes, inconsistent outcomes, and moderate-to-high risk of bias in several studies limit the certainty and generalizability of the evidence. Furthermore, the short follow-up durations underscore the need for larger-scale, long-term RCTs to validate their therapeutic potential and clarify whether benefits extend beyond blood pressure reduction to include meaningful improvements in functional outcomes. Future research should also prioritize standardized dosing, intervention duration, and outcome measures to enhance comparability across studies.
